# Tumors of the Maxilla*Abstract of Paper read before the Dental Section, Amer. Med. Association, Philadelphia,, June, 1897.

**Published:** 1897-08

**Authors:** William Knight

**Affiliations:** Professor of Anatomy and Oral Surgery, Ohio College of Dental Surgery, Cincinnati, Ohio


### THE DENTAL REGISTER.

VOL. LI.] AUGUST, 1897. [No. 8.

### COMMUNICATIONS.

### Tumors of the Maxilla.*

BY WILLIAM KNIGHT, M.D., D.D.S.,

Professor of Anatomy and Oral Surgery, Ohio College of Dental Surgery, Cincinnati, Ohio.

In this paper it is intended to briefly describe some of the more ordinary neoplasms of the maxilla.

The upper as well as the lower maxillary bones, besides being subject to diseases affecting other bony structures of the body, are liable to the invasion of neoplasms, peculiar and limited to themselves, among these may be mentioned the epulides, dentigerous and multilocular cystic tumors. The pathological congenears of the epulides, may, however, be found in other parts of the body, but it is not customary to call every growth found arising from the gums or from the periosteum of the alveolus epulus. Two distinct forms of epulus, and only two forms, are to be recognized as affecting these bones. The recognition of these two distinct forms of epulus is of importance not only from a pathological, but mainly from a surgical standpoint, as deciding the proper treatment which is different in two affections. The hard or fibrous form is by far the most innocent in its pathological effects; it is almost identical with the fibrous growths found in other parts of the body, yet it differs from them mainly in its tendency to form spicula of bone in its construction.

The myeloid or softer form is composed of but a small amount of fibrous tissue, the rest of the neoplasm being largely composed

*Abstract of Paper read before the Dental Section, Amer. Med. Association, Philadelphia,, June, 1897.

of polynucleated myeloid cells, the first variety is closely connected with the gum, also with the periosteum of the alveolus, it springs from the inner or outer surface and unless of long standing and of large growth, seldom if ever invades the entire thickness of the alveolar process. The second form, or myeloid variety, is more closely allied to the endosteal than the periosteal structures, and is connected with the alveolus. It has a marked tendency to invade the entire thickness of the alveolar process; it does not attack the deeper portion of the bone unless improperly treated with irritants, when it may assume a malignant condition and quickly infiltrate the surrounding tissues.

The etiology of either form of epulus is somewhat obscure, both varieties have been observed before the eruption of the teeth of infants. It is, however, generally agreed that some prolonged dental irritation is the usual exciting cause of this affection as instanced in the following case: A lady, aged forty-five, was referred two years ago, to me for treatment. She had worn for five years a lower dental plate that had caused her some annoyance, yet was unattended by any special pain. On June 10th, 1893, when I examined her mouth, there was seen a small growth the size of a hazel nut, occupying the bicuspid region of the left lower jaw; she stated that the growth had been present for more than a year, but to her great alarm had enlarged considerably during the past few months. Having consented to an operation, the alveolar process from the lower lateral incisor to the first molar down to, and including, a portion of the basilar part of the bone was removed, when a fragment of the root of the second bicuspid was found embedded in the bones. This fragment of a tooth has been in all probability the original cause of the formation of the tumor, which upon section, proved to be a myeloid growth. Although the jaw was accidentally fractured toward the completion of the operation, no untoward symptom followed. At the present date, May 31st, 1897, the patient is wearing a new lower plate. There is no evidence of any return of the disease.

In all instances in which the surgeon suspects a myeloid epulus, he should not rest content without removing the entire thickness of the alveolar process. There is perhaps no more pitiful story in surgery than for a surgeon, when a case of the kind returns to him with a sarcoma occupying the seat of the former operation, with perhaps infilteration of the floor of the mouth, to declare that permanent relief is almost hopeless, and to think, if during the operation, more of the jaw had been removed this might not have happened. The essayist speaks from experience, and he hopes never again to make the same mistake.

In the treatment of myeloid epulus, in consequence of the extensive involvement of the alveolar portion of the bone, when the growth apparently springs from one plate only, radical measures must be insisted upon. It is always the wiser plan to remove the entire thickness of the process, sacrificing whichever tooth it may be deemed necessary, to effect a thorough removal of the disease. It is in these cases that imperfect operations are more than useless, for when imperfectly removed it is the unfortunate tendency of myeloid epulus to return in a still more malignant form. This fact is so well recognized that no operator in treating these cases should be satisfied unless he removes outlying tissue beyond the apparent extent of the growth. The best method to be employed for the purpose, is, to saw completely through the alveolar process on each side of the disease and then with one of the various forms of cross-cutting bone forceps, to remove the entire piece of bone with the tumor. It is my invariable practice after having removed the affected piece of bone, to use the bone gouge freely so as far as possible to remove every vestige of disease. The free hemorrhage which arises in these cases is easily controlled by pressing a few small pledgets of cotton in the wound. The hemorrhage having ceased compresses of cotton saturated with a weak boracic acid, or a permanganate of potash solution can be gently pressed into the wound. These compresses are readily retained in position without much inconvenience to the patient, by his simply keeping his mouth closed ; external bandaging is seldom required.

Twenty-four hours having elapsed since the operation, the compresses may be removed and the mouth should be rinsed four times a day with a solution of five grains of permanganate of potash to one ounce of water. In ordinary cases no other treatment is required.

In the fibrous or hard form of epulus, it is seldom necessary to remove the entire thickness of the alveolar process, although it is desirable always to remove either the inner or outer plate upon which the growth is attached. It is prudent in order to effect a limited destruction of the bones, to touch the site of the removed growth with the actual cautery. Those who object to the cautery can produce the same result, by touching the part with nitric acid, chloride of zinc, or the trichlorate of acetic acid. It is seldom necessary to sacrifice the teeth contiguous to the tumor as the unremoved plate is sufficient to retain them in place, and in many instances, years after the operation, the teeth are found to to be firm and in good condition. Cystic diseases of the maxillary bones are perhaps more frequently due either to diseases oi fully developed teeth or they are associated with teeth that have been imperfectly developed. The formation of cysts from either one of these two causes may attain to a very large size, giving rise to absorption to a greater or less extent of the walls of maxilla, without in so doing giving rise to much inconvenience to the patient. It is claimed by Broca, whose views on this subject meet with general recognition, that the great majority of cysts of the jaws have their origin in tooth follicles. It is held that the soft gelatinous enamel organ being easily affected by morbid influences readily disappears, leaving in the follicle a cavity which is ready to be transformed into a cyst. Some of these cases seem to arise without any appreciable cause; an instance of this kind was four years ago referred to me by Dr. F. Sage, of Cincinnati. The lady, forty-six years of age, had, occupying the right side of the lower jaw, a small cystic tumor in the bicuspid region; the tumor had been of slow but continuous growth; at no time during the development had pain been experienced, she sought medical counsel partly on account of the inconvenience of its increasing size, but mainly through the fear that so often possesses the laity, that whenever a growth appears on any part of the body, it must of necessity be a cancer. Careful inquiry into her history failed to elicit any fact that would give a clew to the cause of the trouble upon which an opinion might be founded. Both dentitions had been normal and the teeth in the immediate neighborhood of the growth were in good condition. A careful search was made during the operation to discover any probable cause that might have produced a tumor, but none was found. Four years have passed since the operation, and there has been no return of the affection. Although the etiology of cystic tumors occurring in the jaw bones has in recent years been elucidated to a very large extent, much still remains in doubt.

Mr. Christopher Heath, in his work on the "Diseases of the Jaw," has made particular mention of the views held by *Mr. F. Eve, as to the development of multilocular cystic tumors of the maxilla. Mr. Eve maintains, that so far from multilocular cystic tumors having a dental origin, they are, on the contrary, produced by an ingrowth of the epithelium of the gum. This class of tumors frequently follow injury or irritation of decayed teeth or long continued inflammation, which has led to an increased supply of blood to the parts. They are slow of growth, they do not affect the lymphatic glands, nor infiltrate the surrounding parts.

These tumors are innocent in character, but owing at times to their enormous increase and multiplicity, they not unfrequently necessitate the removal of the larger portion of the lower jaw, which bone is especially obnoxious to the disease.

The treatment of cystic humors necessarily varies according to the form of the cyst; if it be a single cyst connected with the fang of a tooth, the extraction of the offending body will usually be sufficient to effect a cure. If, however, it be of larger size and has caused an expansion of the bone, an incision through its bony wall will be necessary; this operation will evacuate the fluid which is of a dark, clear color, unless inflammation has been present, in which event the contents will be of a more purulent character. It is also the best practice to cut away a good portion of the cyst wall as otherwise the cyst is likely to rapidly reform. In all

*Mr. Eve's paper as originally published is to be found in the *British Medical Journal* for Jan. 6th, 1893.

instances in which a tooth is suspected of being present within a cyst, a careful search for it should be made for it is obvious that the solution will be impossible so long as the tooth is retained. In both of the above instances, the after-treatment consists in simply using, four times a day, an antiseptic and slightly astringent mouth-wash. In these cases when a portion of the wall of the cyst has been removed, the cavity should be plugged with boracic acid gauze or lint that has been saturated with a solution of permanganate of potash. This packing might be removed and a fresh one used if necessary in forty-eight hours time.

But in the treatment of multilocular cystic tumors, more radical measures must be adopted. If any suspicion exists as to the possibility of a sarcomatous complication, which is not infrequently the case in this character of growth, the removal of the portion of the affected bone is always recommended and has been the usual procedure. If, however, the diagnosis can be satisfactorily determined as to the absence of a malignant factor the operation as recommended by Mr. Butcher, of Dublin, can be performed. This operation consists in dividing the mucous membrane covering the cyst, and then with gouge and bone forceps, or chisel and mallet, thoroughly removing all the affected parts. The success of this operation depends entirely upon the completeness with which this is done.

A malignant tumor composed of the same structural elements differs in its clinical history according as it attacks the soft or the bony tissues and differs also in bone as to whether the compact or cancellous tissue is involved, thus a round-celled sarcoma will not unfrequently develop within the interior of the lower jaw and will give rise to no other symptom than perhaps enlargement of the bone. This condition may continue to exist for several years before the tumor has attained the size of a walnut, the patient during all this period complaining only of a small deformity, caused by the slowly growing enlargement. What a contrast this offers to the rapidly growing sarcoma, as it occurs in the softer tissues. This difference of growth in the same structurally-formed neoplasm may partly be explained by its elementary formation. All the varieties of the sarcoma belong to the type of the embryonic connective tissue, the cells of which they are mainly composed have no definite arrangement; they are not in constant relation with the sarcoma and consequently the cell not being surrounded by alveola as in the case with carcinomata has a marked tendency to infiltrate the neighboring tissues at a very early period. The blood vessels of a sarcoma ramify among the cells and are very thin walled, hence the frequent occurrence of hemorrhage into their substance, and their rapid dissemination through the vascular and not the lymphatic system of vessels.

The compact tissue of bone antagonizes, to a marked degree, the favorable conditions of sarcomatus growth, this is especially the case when a sarcoma developes between two plates of bone, as when a neoplasm of this character forms in the interior of the lower jaw. Occupying an intermediary position, chemically speaking, between the rapidly growing sarcoma of the soft parts, and those slow growing ones occurring in the lower jaw, are those instances of sarcoma originating in the connective tissue of the mucous membrane lining the antrum of Highmore. This is by no means an uncommon location for the development of primary sarcoma. The history of all such cases is very similar. The sufferer complains of pain which is often referred to a tooth contained in the affected jaw. The offending member is sometimes treated, more frequently, however, it is extracted, the relief, if any, afforded by these means is but temporary; the uneasiness and pain continued, with perhaps some intermission. This condition may, and usually does continue for a considerable time before any swelling of the part is observed; after the swelling appears, the disease is apt to increase rapidly, and the growth, which by this time has completely filled the antrum, and has infiltrated the bony walls of the cavity, has a tendency to fungate and to bleed readily. A case of this kind was referred to me by Dr. F. Saul. The sufferer was a man forty-two years of age. For two years past he had intermittent pain in the upper second bicuspid. Although the tooth was in sound condition, he had it extracted, yet, without obtaining any relief. Two months subsequently when he came to me, a slight swelling appeared on the right cheek, and a suspicious looking growth in the alveolus of the extracted tooth. He stated that he had some pain almost all the time which now and then was severe. He was advised to have the right maxillary removed ; to this however he dissented. Five months subsequently he returned. The swelling now extended upward under the zygoma and reached the temporal region. To operate with hope of permanent relief, under such conditions, being extremely doubtful, he was so informed, as he was now very eager to have the operation performed. The upper right half of the maxillary bone was removed, together with considerable of the surrounding tissue. The tumor proved to be a round-celled sarcoma. Seven months after, the disease returned and a second operation was performed. This, for a few months, gave him relief, after which time hemorrhage ensued, which occurring periodically, terminated in death.

To a certain extent this case illustrates the clinical history of sarcoma when originating in the antrum. Intermittency of growth at first, which is somewhat characteristic of sarcoma wherever it occurs. This was followed by slow but continuous enlargement, and finally after the bony walls of the antrum had yielded by a rapid increase in growth with infiltration of all the surrounding parts. The treatment of sarcoma must be radical. Any contemplated operation should be done as early as a diagnosis can be made. As long as a sarcoma is confined within the bony walls of the antrum, a hopeful result as to a permanent cure, can be looked for, but after this disease has passed beyond these confines, any operation, however heroic it may be, will result usually in failure, as it is almost impossible to remove all the affected tissues. As for myself, I always refuse to operate in advanced cases of sarcoma of the upper jaw without informing the patient or his friends of a probability of a return of the disease and agreeing to operate simply as a means of relieving temporarily the suffering of the person so affected.

---

LINSEED oil is a good thing for corns. A piece of lint dampened with the oil should be wrapped round the part and kept constantly applied. It gives great relief where the corn is soft, and is not long in eradicating it.

